# Hypothermia-Associated Coagulopathy: A Comparison of Viscoelastic Monitoring, Platelet Function, and Real Time Live Confocal Microscopy at Low Blood Temperatures, an *in vitro* Experimental Study

**DOI:** 10.3389/fphys.2020.00843

**Published:** 2020-07-14

**Authors:** Bernd Wallner, Bettina Schenk, Martin Hermann, Peter Paal, Markus Falk, Giacomo Strapazzon, Wenjun Z. Martini, Hermann Brugger, Dietmar Fries

**Affiliations:** ^1^Department of Anaesthesiology and General Intensive Care Medicine, Medical University of Innsbruck, Innsbruck, Austria; ^2^Department of General and Surgical Intensive Care Medicine, Medical University of Innsbruck, Innsbruck, Austria; ^3^Institute of Mountain Emergency Medicine, Eurac Research, Bolzano, Italy; ^4^Department of Anaesthesiology and Intensive Care Medicine, Hospital of the Brothers of St. John of God, Paracelsus Medical University, Salzburg, Austria; ^5^U.S. Army Institute of Surgical Research, Fort Sam Houston, San Antonio, TX, United States

**Keywords:** coagulation, hypothermia, platelet, platelet activity, fibrinogen, viscoelastic testing, Confocal Microscopy

## Abstract

**Introduction:**

Hypothermia has notable effects on platelets, platelet function, fibrinogen, and coagulation factors. Common laboratory techniques cannot identify those effects, because blood samples are usually warmed to 37°C before analysis and do not fully reflect the *in vivo* situation. Multiple aspects of the pathophysiological changes in humoral and cellular coagulation remain obscure. This *in vitro* experimental study aimed to compare the measurements of thromboelastometry (TEM), multiple-electrode aggregometry (MEA) and Real Time Live Confocal Imaging for the purpose of identifying and characterizing hypothermia-associated coagulopathy.

**Methods:**

Blood samples were drawn from 18 healthy volunteers and incubated for 30 min before being analyzed at the target temperatures (37, 32, 24, 18, and 13.7°C). At each temperature thromboelastometry and multiple-electrode aggregometry were measured. Real Time Live Confocal Imaging was performed at 4, 24, and 37°C. The images obtained by Real Time Live Confocal Imaging were compared with the functional results of thromboelastometry and multiple-electrode aggregometry.

**Results:**

Thromboelastometry standard parameters were impaired at temperatures below baseline 37°C (ANOVA overall effect, *p* < 0.001): clotting time was prolonged by 27% at 13.7°C and by 60% at 18°C (*p* < 0.044); clot formation time was prolonged by 157% (*p* < 0.001). A reduction in platelet function with decreasing temperatures was observed (*p* < 0.001); the area under the curve at 13.7°C was reduced by 96% (ADP test), 92% (ASPI test), and 91% (TRAP test) of the baseline values. Temperature-associated changes in coagulation were visualized with Real Time Live Confocal Imaging. Molecular changes such as the temperature-associated decrease in the fibrin network are paralleled by cellular effects like the lesser activity of the platelets as a result of decreased temperature. The maximum clot firmness (MCF) in TEM only changed slightly within the temperature range tested.

**Conclusion:**

The inhibitory effects of temperature on clot formation were visualized with Real Time Live Confocal Microscopy and compared with standard point-of-care testing. Inhibition of clotting factors and impaired platelet function are probably a result of hypothermia-induced impairment of thrombin. Measurement of MCF in TEM does not fully concur with Real Time Live Confocal Microscopy or MEA in hypothermia.

## Introduction

Humans have survived extremely profound hypothermia. The lowest recorded core body temperature caused by accidental hypothermia that was survived neurologically intact is 13.7°C (Gilbert et al., 2000). Case series from 1961 describe patients who survived induced hypothermia of 4.2°C without neurological impairment (Stephen et al., 1961). Survival in traumatic cardiac arrest patients after exsanguination is poor and hypothermia in trauma patients is associated with increased mortality (Wang et al., 2005; Moffatt et al., 2018). Contrarily, experimental research is focusing on the concept of “Suspended Animation” where profound hypothermia rapidly induced by cardiopulmonary bypass in severe trauma resulting in delayed resuscitation improved survival and preserved neurological function (Moffatt et al., 2018; Mohiyaddin et al., 2018).

Trauma-related hypothermia has notable effects on the coagulation system and is an independent risk factor for increased morbidity and mortality in trauma patients (Jurkovich et al., 1987; Wolberg et al., 2004; Gerecht, 2014; Vardon et al., 2016). Trauma-induced hypothermia with a core temperature <32°C has been associated with 50–100% mortality (Jurkovich et al., 1987; Wang et al., 2005). In mild hypothermia (35–32°C), bleeding results primarily from a defect in platelet adhesion, and at <33°C enzyme activity contributes additionally to coagulopathy (Wolberg et al., 2004).

Management of hypothermia-associated coagulopathy is challenging. The effects of hypothermia on humoral and cellular coagulation cannot be identified with standard laboratory techniques such as platelet count, prothrombin time (PT), activated partial thromboplastin time (aPTT), or activated clotting time (ACT), as they do not reflect the *in vivo* situation of low blood temperatures because samples are usually warmed to approximately 37°C for analysis (Martini et al., 2008). Rotational thromboelastometry (TEM) (i.e., ROTEM^®^) is a point-of-care diagnostic tool with the ability to measure the variables at the body temperature of the patient (Johansson et al., 2009; Park et al., 2009; Schochl et al., 2010). A previous study using TEM demonstrated that hypothermia (25°C) progressively impairs coagulation (Rundgren and Engstrom, 2008). That study stated that coagulation time (CT) and clot formation time (CFT) were significantly prolonged, but maximum clot firmness (MCF) was the variable least significantly impaired (Rundgren and Engstrom, 2008). Another study performed at wide temperature range testing with ROTEM coagulation analyses has shown that MCF was unchanged at different temperatures for all tests while significant changes in CT, CFT, and alpha angle were found (Kander et al., 2014). MCF, among others, reflects changes in the number of platelets. The detrimental effects on platelet function, on the contrary, are well-established and have been demonstrated with multiple-electrode aggregometry (MEA) (i.e., Multiplate^®^) in mild and moderate (32–28°C) hypothermia (Mueller et al., 2007; Scharbert et al., 2010).

Real Time Live Confocal Imaging allows visualization of blood coagulation under different circumstances (Weber et al., 2015). Beside platelets as the cellular components of coagulation, fibrin (Weiss et al., 2017) and Factor XIII can be visualized using Real Time Live Confocal Imaging (Weber et al., 2018). To our knowledge, no study has reported the comparisons of functional coagulation methods such as TEM and MEA with optical methods like Real Time Live Confocal Imaging.

This *in vitro* experimental study aimed to identify and characterize hypothermia-associated coagulopathy. Images of blood clotting obtained with Real Time Live Confocal Microscopy were compared with quantitative measurement using TEM and MEA.

## Materials and Methods

### Ethics Approval

This study was approved by the human subjects review board of the Medical University of Innsbruck, Austria (Ref. No. AN2016-0102 362/4.13). Written informed consent was obtained from all study participants. The study was performed in compliance with the Declaration of Helsinki and followed the Good Clinical Practice guidelines as defined by the International Conference on Harmonization (ICH-GCP).

### Population

Healthy randomly selected volunteers (*n* = 18, 9 females, 22–46 years) were enrolled at the Department of Anesthesiology and General Intensive Care Medicine, Medical University of Innsbruck, between January and May 2017. Exclusion criteria were pregnancy, breastfeeding, active participation in another clinical trial, any form of medication interfering with coagulation, or known coagulopathies of the participant.

### Collection and Preparation of Blood Samples

Blood samples were drawn into vacutainer tubes containing 1/10 volume of 3.12% trisodium citrate (Sarstedt, Nümbrecht, GER) for TEM and fibrinogen measurements. Hirudin tubes (Sarstedt, Nümbrecht, Germany) were used for platelet function testing with a Multiplate^®^ Analyzer (Roche Diagnostics, Mannheim, Germany). The various blood samples were incubated for 30 min before being analyzed at the target temperatures. Target temperatures were physiological core body temperature (37.0°C) and the three cut-off values for accidental hypothermia stages (32, 24, and 18°C) as well as the lowest temperature ever accidentally acquired and survived (13.7°C) (Gilbert et al., 2000; Paal et al., 2016). The analyses were performed starting with the lowest temperature and ending with the highest temperature in order to minimize the confounder of platelet storage.

### Thromboelastometry

The ROTEM^®^ (Innovations GmbH, Munich, Germany) temperature was set to the target temperature of the blood samples. After incubation and within 4 h after blood draw the samples were analyzed with the extrinsically activated assay with tissue factor (ExTEM). Standard parameters were evaluated including coagulation time (CT), i.e., the time until clot formation starts; clot formation time (CFT), i.e., the time from CT until the clot reaches an amplitude of 20 mm; A5 value (A5), indicating the clot amplitude 5 min after CT. A5 is a tool for fast detection of changes in coagulation; maximum clot firmness (MCF), defined as the maximum amplitude reached by clot formation during measurement; and alpha angle (alpha), which is the angle between the middle axis and the tangent to the clotting curve through the 20 mm amplitude point.

### Platelet Function Testing

Hirudin whole blood samples were incubated at the respective target temperature, as mentioned above. Subsequently and within 4 h after blood draw, blood samples were analyzed using a Multiplate^®^ Analyzer (Roche Diagnostics, Basel, CH) and initiated using arachidonic acid 0.5 mM (ASPI test), thrombin receptor-activating peptide-6 32 μM (TRAP test), or adenosine diphosphate 6.4 μM (ADP test) according to the manufacturer’s instructions. Test results were expressed as the area under the curve (AUC). Device temperature was set to the respective experimental approach (13.7–37°C, see above).

### Real Time Live Confocal Microscopy

Citrated whole blood samples were incubated at the respective target temperature, as mentioned above. The temperature of the specimen was meticulously monitored and managed during the entire duration of the experiment, with the exception of confocal imaging, which was less than 1 min. Nevertheless, the room in which the microscope is situated was precisely controlled to match the temperature of the specimen. Visualization of fibrin networks via the addition of fluorescein isothiocyanate-linked fibrin-binding peptide (Weiss et al., 2017) under static conditions was performed in Lab-Tek 8-well chambered #1.0 borosilicate cover glass slides (Nunc, Rochester, NY, United States). For this purpose, 200 μl of either citrated plasma or blood were pipetted into each well. Coagulation was induced via addition of 5 μl star-tem (0.2 mol/l CaCl2 in HEPES buffer pH 7.4) and 5 μl ex-tem (recombinant tissue factor; both reagents from Tem Innovations GmbH, Basel, CH). The fibrin network as well as the FXIII activity were visualized by adding iFXIIIa (final concentration 0.5 ng/μl; GenicBio Limited, Shanghai, CN), as described elsewhere (Jaffer et al., 2004; Weber et al., 2018). The cellular components such as platelets and erythrocytes were stained with wheat germ agglutinin-alexa fluor 555 (WGA, final concentration 5 μg/mL; Thermo Fisher Scientific, Waltham, MA, United States). Incubation time for both stains was 15 min at the temperatures of 4, 24, and 37°C. Real Time Live Confocal Imaging was performed with a spinning disk confocal system (UltraVIEW VoX; Perkin Elmer, Waltham, MA, United States) connected to a Zeiss AxioObserver Z1 microscope (Zeiss, Oberkochen, Germany). Images and z stacks were acquired using Volocity software (Perkin Elmer, Waltham, MA, United States) using a 63× oil immersion objective with a numerical aperture of 1.42. Z stacks of 10 μm are shown with an optical spacing of 1 μm.

### Statistics

Sample size estimation for this trial was based on experimental data described elsewhere (Ruzicka et al., 2012). We calculated that 18 participants would provide a power of 80% at a 5% significance level to determine the prespecified effect size of 80%, assuming a 10% dropout rate and 10% security margin. Data are presented as mean and standard deviation or frequencies, as appropriate. Differences in temperatures were assessed with an analysis of repeated measures (ANOVA) using a mixed model with temperature as repeated effect assuming an unstructured covariance matrix, followed by Bonferroni-corrected *post-hoc* tests. Normality at 37°C was assessed visually and by means of Shapiro-Wilk test. *P*-values are two-sided and values <0.05 were considered statistically significant and SPSS 21 (IBM, Armonk, NY, United States) was used for analysis.

## Results

### Population Characteristics

A total of 18 volunteers participated in this study, nine of whom were women and nine men with a mean age of 31 years (range 22–46 years), height of 174 cm (range 158–186) and weight of 65 kg (range 50–95). Examination of basic coagulation values under regular standard laboratory conditions at a temperature of 37°C (PT, aPTT, INR, fibrinogen, thrombin time, AT III) and platelet count showed no pathological deviation in any participant. Volunteer characteristics are displayed in Table 1.

**TABLE 1:** Demographic data (*N* = 18).

	Median	Min	Max	Mean	*SD*
Age (years)	31	22	46	31	7
Height (cm)	174	158	186	173	8
Weight (kg)	65	50	95	67	11
BMI (kg/m^2^)	21.6	20.0	29.3	22.1	2.2

**SD, standard deviation.**

### Thromboelastometry

**FIGURE 1:**
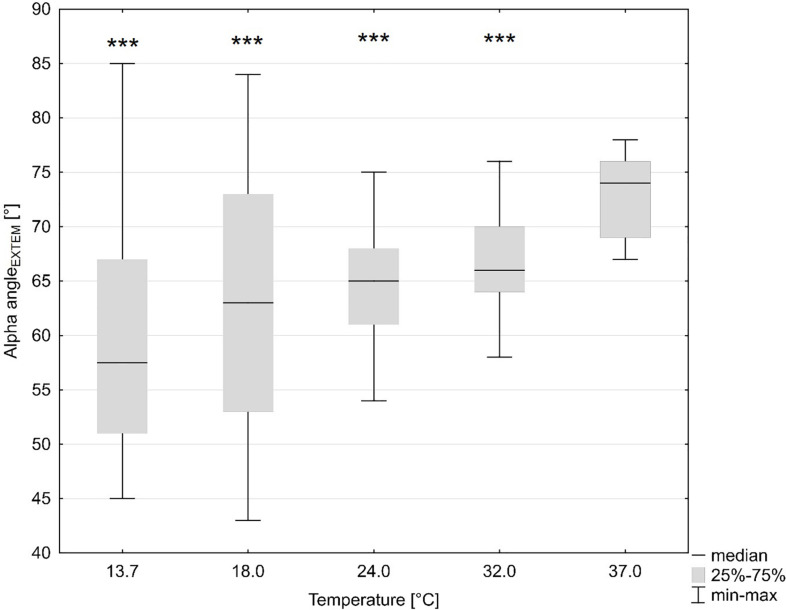
Influence of temperature (x axis, temperature in degrees Celsius) on global coagulation (y axis, alpha angle in ExTEM in degrees). ***p < 0.001 for significant differences as assessed with repeated measures ANOVA as compared to baseline (37°C).

TEM standard parameters were impaired at temperatures (ANOVA overall effect, *p* < 0.001 for all) below baseline (37°C): at the lowest temperature (13.7°C) CT was prolonged by 27% of the baseline values (not significant) and at 18°C by 60% (*p* < 0.044, Bonferroni-corrected). Accordingly, the A5 value dropped by 35% with decreasing temperature (at 13.7°C) as compared to baseline (*p* < 0.001, Bonferroni-corrected). CFT was prolonged by 157% (*p* < 0.001, Bonferroni-corrected), while MCF was slightly but significantly reduced by 4% (*p* = 0.006, Bonferroni-corrected), and the alpha dose-dependently reduced by up to 19% (*p* = 0.001, Bonferroni-corrected) (Figure 1) at the lowest temperature (Table 2).

**TABLE 2:** Laboratory parameter.

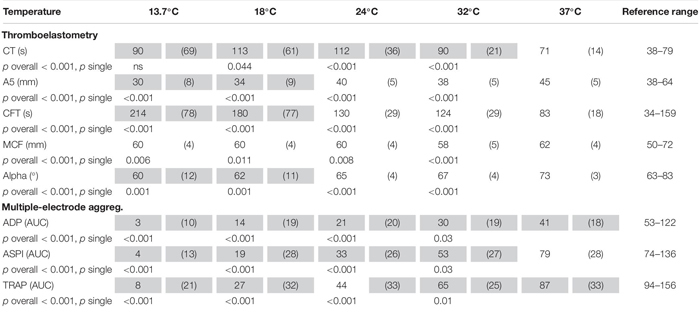

**Mean values (standard deviation) of standard parameters of thromboelastometry and multiple-electrode aggregometry for the various temperatures. Values outside the reference range are color-marked. The p-value for the overall temperature effect as well as the Bonferroni-corrected p-values for single comparisons between the baseline and the respective temperature are given below each parameter. A5, clot amplitude 5 min after CT; ADP, adenosine diphosphate; alpha, alpha angle; ASPI, aspirin; AUC, area under the curve; CFT, clot formation time; CT, coagulation time; MCF, maximum clot formation; TRAP, thrombin receptor-activating peptide.**

### Multiple-Electrode Aggregometry

**FIGURE 2:**
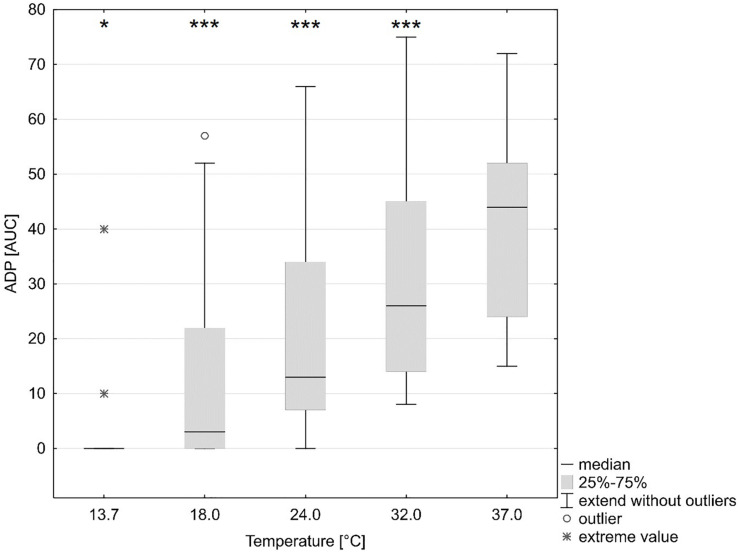
Influence of temperature (x axis, temperature in degrees Celsius) on platelet function (y axis, ADP test multiple-electrode aggregometry, AUC area under the curve). ^∗^*p* < 0.05, ^∗∗∗^*p* < 0.001 indicates differences as assessed with the repeated measures ANOVA as compared to baseline (37°C). Figure shows median, 25 and 75% quartile, extended values, outliers and extreme values.

A reduction in platelet function with decreasing temperatures was observed (ANOVA overall effect, *p* < 0.001, Bonferroni-corrected, Table 2). The AUC was reduced by 96% (*p* < 0.001, Bonferroni-corrected, ADP test, Figure 2 and Table 2), 92% (ASPI test), and 91% (TRAP test) as compared to baseline (37°C).

### Real Time Live Confocal Imaging

**FIGURE 3:**
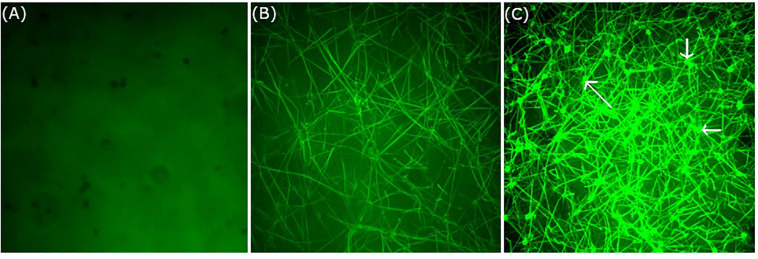
Influence of temperature on coagulation and platelet function visualized with Real Time Live Confocal Imaging. Representative pictures of one healthy volunteer were chosen to visualize the fibrin net (green, iFXIIIa) at **(A)** 4°C, **(B)** 24°C, and **(C)** 37°C. Note the near absence of a fibrin network at 4°C and the highest density of the fibrin network under 37°C conditions as compared to the fibrin network formed at 24°C. This is also paralleled by platelet activity as shown in C (see arrows). Objective: 63× oil immersion.

**FIGURE 4:**
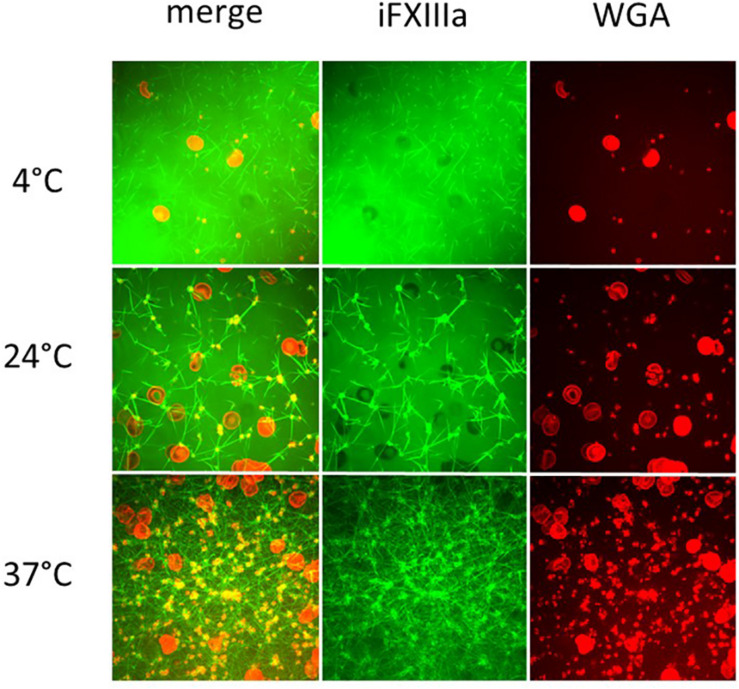
Influence of temperature on coagulation and platelet function visualized with Real Time Live Confocal Imaging. Representative pictures of one healthy volunteer were chosen to visualize the fibrin network (green, iFXIIIa), platelet aggregates (yellow due to WGA and iFXIIIa overlay), and erythrocytes (red, WGA) at 4°C (first row), 24°C (middle row), and 37°C (bottom row). Merged channels (left column) as well as iFXIIIa channel (middle column) and WGA channel (right column) are shown. As expected, fibrin network density increases with temperature as well as platelet activity. Objective: 63× oil immersion.

Temperature-associated changes in coagulation and platelet function were visualized with Real Time Live Confocal Imaging (Figures 3, 4). Molecular changes such as the temperature-associated decrease in the fibrin network were paralleled by cellular effects such as the lesser activity of the platelets as a result of decreased temperature. There was near absence of a fibrin network at 4°C and the highest density of the fibrin network under 37°C conditions as compared to the fibrin network formed at 24°C and impaired platelet activity.

## Discussion

This study compared the reliability of Real Time Live Confocal Imaging as a new tool for the diagnosis and visualization of hypothermia-associated changes in coagulation and the reliability of the established analytic methods like TEM and MEA. The inhibitory effects of temperature on clot formation were visualized with Real Time Live Confocal Microscopy in this study and compared with standard point-of-care testing. Real Time Live Confocal Imaging confirms the disappearance of a strong clot network and platelet activity during blood cooling. Inhibition of clotting factors and impaired platelet function are probably a result of hypothermia-induced impairment of thrombin. Currently established analytic methods like TEM do not fully concur with Real Time Live Confocal Microscopy and omit the effect on platelets and platelet activity by displaying unchanged MCF values. TEM and MEA only partially confirm coagulopathic changes caused by impairment of the clot network during blood cooling and demonstrate an absence of coagulative activity at the lowest temperatures, probably as a result of hypothermia-induced impairment of thrombin.

In an attempt to systematically study the effects of various stages of hypothermia on the coagulation system, this study was designed as an *in vitro* study using the three methods (Real Time Live Confocal Imaging, TEM, and MEA) to investigate whole blood from healthy volunteers. Real Time Live Confocal Imaging images indicate a significant correlation between temperature and pathological changes in cellular as well as humoral components of coagulation. In parallel, the study also included TEM and MEA, which have been employed for the systematic analysis of coagulation in previous studies (Rundgren and Engstrom, 2008; Jeppesen et al., 2018).

Real Time Live Confocal Imaging allows novel insights into non-fixed plasma samples in great detail and resolution. Due to our short exposition times (900 ms) and the low laser power of 5% temperature changes can be neglected. It is known that coagulation can be induced by laser exposition as described in a review (Norman, 2005). However, much higher energy levels have to be applied for such a purpose. Induction of coagulation as well as staining and incubation were performed at 4, 24, and 37°C, followed by immediate imaging. We therefore believe, that for this purpose and approach a temperature bias is insignificant. The clear differences in the coagulation, visualized by real time live confocal imaging, show the temperature dependence on a cellular as well as molecular level.

In addition to imaging molecular key players (fibrin) and coagulation factor activity, Real Time Live Confocal Imaging can also visualize the influence of hypothermia on platelets and erythrocytes. While platelet activity is hardly detectable at low temperatures, it can be observed at 24°C, although notably less strong than at 37°C. The best platelet activity was measured at 37°C. At temperatures <24°C platelets show decreased movement and impaired reactivity. Platelets observed at temperatures <24°C lack activation and the formation of pseudopodia. This effect prevents enlargement of the platelets’ surface and fibrin-mediated connection to other platelets. For this pilot study without any previous data on Real Time Live Confocal Imaging on hypothermic coagulation, we aimed to test the extreme conditions in temperature physiology. For this purpose, we selected 4°C as the lowest target temperature at which we would evaluate this extreme form of hypothermia. In the current literature, there is an increasing body of evidence on suspended animation, where severely wounded patients are cooled after exsanguinating hemorrhage during emergency surgery (Moffatt et al., 2018; Mohiyaddin et al., 2018). Case series from 1961 describe patients who survived induced hypothermia of 4.2°C without neurological impairment (Stephen et al., 1961). For technical reasons, the modes of cooling the blood were different from those employed for TEM and MEA, where 4°C could not have been tested in a reproducible manner. Coagulation function and platelet function have been reported to vary with individual conditions, such as age, gender, diabetes, obesity, cholesterol levels and chronic diseases (Wang and Tall, 2016). Although we did not stratify our normal subjects accordingly in this pilot study, the methodology of Real Time Live Confocal Imaging used in the study may facilitate future effort to investigate individual differences and elucidate the underline mechanisms contributing to the differences, as well as the individual responses to hypothermia. An *in vitro* study comparing coagulation at 36°C and at 32°C using TEM confirmed that coagulation was impaired at 32°C (Kettner et al., 2003). In another study assessing coagulation with TEM at a temperature range from 25 to 40°C, the authors found a CT prolongation of 121% and a CFT prolongation of 220% at 25°C as compared to 40°C (Rundgren and Engstrom, 2008). The same study found that MCF, primarily assessing platelet function, was the variable least significantly impaired and the maximum impairment was 8%, namely much less inhibited than CT, CFT, or alpha (Rundgren and Engstrom, 2008). The present study confirmed those findings and showed that CT, CFT, and alpha as measured by TEM were increasingly impaired with decreasing temperatures. This study also found that MCF was not considerably reduced despite a remarkable reduction in the fibrin network as shown by Real Time Live Confocal Imaging. This was in accordance with previous studies and can be explained by the fact that TEM measures the viscosity of the blood and the clot. Cooled blood demonstrates a higher viscosity and TEM, as a viscometer, measures false normal values, especially in MCF.

A direct comparison of thrombocyte function in Real Time Confocal Microscopy under hypothermic conditions shows significant impairment at low temperatures. In our study, MEA was used to analyze the temperature-associated effect on platelets. We found a significant correlation between low temperatures and platelet dysfunction. It was thus reasonable to correlate the qualitative image obtained with Real Time Live Confocal Imaging, which shows immobile and inactive platelets, with the linear decrease in platelet function in MEA. This result of the current study, which is in agreement with previous studies, has significant implications for daily practice (Mueller et al., 2007; Scharbert et al., 2010). Diagnostic measures like TEM are more frequently used, also as point-of-care testing, and are being integrated into algorithms for the treatment of massive hemorrhage and multiple-trauma patients. When only looking at CT and MCF for clinically important parameters, significant impairment of thrombocytes could be missed, with implications for management and treatment of hypothermic trauma patients.

The current literature evidences a controversy concerning platelet function at low temperatures. One study found no changes in the MCF at low temperatures (25°C) and concluded that the hypothermia-associated effect is more likely to be related to humoral coagulation factors (Rundgren and Engstrom, 2008). Wolberg et al. (2004) stated that mainly platelets and tissue factor-bearing cells could potentially be affected by temperature. In addition, platelet aggregation and adhesion were significantly reduced at 33°C by comparison with 37°C. Below 33°C, however, both enzyme activity and platelet function were reduced (Wolberg et al., 2004). Another study found that overall platelet aggregation was increased in mild hypothermia (34°C), as compared to 37°C (Scharbert et al., 2010). At lower temperatures (32–28°C) the quantitative and qualitative platelet abnormalities intensify hypothermia-related coagulopathy (Villalobos et al., 1955; Patt et al., 1988; Meng et al., 2003). Those findings are supported by the current study, which confirmed the profound impairment in platelet function in lower temperatures. Nevertheless, we did not find any form of increased platelet function in moderate hypothermia.

Hypothermia has a significant negative impact on thrombin generation since it primarily inhibits the initiation phase of thrombin generation (Martini, 2009). The physiological effect of thrombin as a promotor of coagulation factors V, VIII, and XI is thus inhibited. Hypothermia also results in a missing stimulus for the activation of platelets. A study performed on survivors after out-of-hospital cardiac arrest treated with targeted temperature management (TTM) confirms that prolonged TTM impairs thrombin generation (Jeppesen et al., 2017). This study parallels the findings of the present study. Even though we did not measure thrombin directly, we indirectly demonstrated the impact of impaired thrombin generation through prolonged CT, a change in the alpha angle and significantly reduced platelet function, as shown by MEA.

A previous study described dynamic changes in the metabolism of fibrinogen during hypothermia. Hypothermia of 32°C primarily inhibits the initiation phase and decreases fibrinogen synthesis as compared to normothermia (Martini, 2007, 2009). Another study established that hypothermia-associated coagulopathy is more likely related to a reduced availability of platelet activators, rather than being a consequence of an intrinsic defect in platelet function (Van Poucke et al., 2014). The present study confirms that both these temperature-associated changes in molecular and cellular components of coagulation were visualized with Real Time Live Confocal Imaging. The confocal images provide a universal overview of impaired cellular and molecular coagulation. Most impressive is the complete disappearance of the clot network at low temperatures (i.e., <24.0°C). The density of the fibrin network increases at 24°C and is highest at 37°C, which is in accordance with previous results (Martini et al., 2008).

However, the decreases in fibrin network formation caused by hypothermia and visualized with Real Time Live Confocal Imaging are inconsistent with the maximum clot firmness as measured with TEM in this study. The different nature of the two measurements may explain the inconsistency. Real Time Live Confocal Imaging provides a snapshot of the fibrin network at certain time points after clotting reagents have been added. Thus, the slower the clotting process is when captured, the looser the clot network appears. The maximum clot firmness reflects the maximum clot strength. The cold-induced slower clotting process may take a longer time to reach maximum strength, but does not affect the maximum strength value, as it is dependent on the concentration of fibrinogen and platelets.

### Limitations

This was an experimental study. The coagulation system was assessed with several state-of-the art methods, which were also calibrated for the target temperatures. In the human body, coagulation is affected by additional factors that cannot be simulated. Secondly, our sample size was limited. Thirdly, different temperature points were used in TEM (13.7, 18, 24, 32, and 37°C) and Real Time Live Confocal Imaging (4, 24, and 37°C) in this study, which made it difficult to compare the two analysis. This limitation was due to technical challenges in changing temperature in the two methods. In TEM, temperature can be changed and maintained conveniently by inputting the designed number within the manufacture range. We did not measure temperature point of 4°C as it is not in the range. In Real Time Live Confocal Imaging analysis, changing operation temperature is difficult, thus less temperature points were used in this study. However, we were able to perform Real Time Live Confocal Imaging at 4°C. Although we were unable to compare the two measurements precisely at each temperature point in this study, we demonstrated the changing patterns of coagulation under hypothermia from the two analysis.

Unfortunately, we made different and fewer temperature points with Real Time Live Confocal Imaging than with other analytic measurements. Real Time Live Confocal Microscopy enables us to analyze extreme hypothermia by cooling the blood to 4°C and obtaining images. Nevertheless, construction of the Real Time Live Confocal Microscope made analyses of various temperature stages difficult. For this reason, we made the measurements at 4, 24, and 37°C.

Precise control of temperatures, on the contrary, can be performed by cooling the thromboelastometer in a climatized room where the experiments were performed. The temperature steps in the thromboelastometer were exactly checked before performing the analyses, but we were not able to cool the room and the thromboelastometer to as low as 4°C.

## Conclusion

The inhibitory effects of temperature on clot formation were visualized with Real Time Live Confocal Microscopy in this study and compared with standard point-of-care testing. Real Time Live Confocal Imaging confirms the disappearance of a strong clot network and platelet activity during blood cooling. Inhibition of clotting factors and impaired platelet function are probably a result of hypothermia-induced impairment of thrombin. Measurement of MCF with TEM does not fully concur with Real Time Live Confocal Microscopy and MEA in hypothermia. Future studies that provide better insight into hypothermia-associated impairment of platelet function and its diagnostics are required.

## Data Availability Statement

The datasets generated for this study are available on request to the corresponding author.

## Ethics Statement

The studies involving human participants were reviewed and approved by the Ethics Committee of the Medical University Innsbruck, Austria. The patients/participants provided their written informed consent to participate in this study.

## Author Contributions

BW contributed to the study conception and design, acquisition of data, analysis and interpretation of data, drafting of manuscript, and critical revision. BS contributed to the study conception, acquisition of data, drafting of manuscript, and critical revision. MH contributed to the study conception and design, performance of Confocal Microscopy, and critical revision. PP contributed to the study conception and design, analysis and interpretation of data, and critical revision. MF contributed to the study conception and design, statistical analysis, and interpretation of data. GS and HB contributed to the study conception and design, acquisition of data, and critical revision. WM contributed to the study conception and design and critical revision. DF contributed to the study conception and design, analysis and interpretation of data, drafting of manuscript, and critical revision. All authors contributed to the article and approved the submitted version.

## Conflict of Interest

The authors declare that the research was conducted in the absence of any commercial or financial relationships that could be construed as a potential conflict of interest.
